# 3-Chloro-5-meth­oxy-2,6-dinitro­pyridine

**DOI:** 10.1107/S1600536809031407

**Published:** 2009-08-15

**Authors:** Jian-feng Guo, Jian-long Wang

**Affiliations:** a School of Chemical Engineering and Environment, North University of China, Taiyuan, People’s Republic of China

## Abstract

In the crystal structure of the title compound, C_6_H_4_ClN_3_O_5_, the two nitro groups are twisted with respect to the pyridine ring, making dihedral angles of 33.12 (13) and 63.66 (14)°.

## Related literature

For the synthesis, see: Bissell & Swansiger (1987 ▶); Chen *et al.* (2008 ▶).
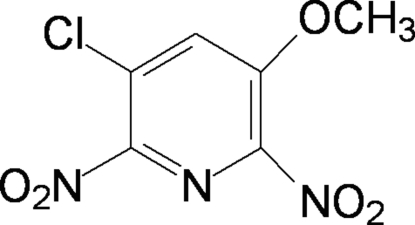


## Experimental

### 

#### Crystal data

C_6_H_4_ClN_3_O_5_
*M* *_r_* = 233.57
Monoclinic, 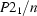

*a* = 6.6490 (13) Å
*b* = 10.842 (2) Å
*c* = 12.715 (3) Å
β = 95.55 (3)°
*V* = 912.3 (3) Å^3^
*Z* = 4
Mo *K*α radiation
μ = 0.43 mm^−1^
*T* = 293 K
0.50 × 0.40 × 0.28 mm

#### Data collection

Rigaku R-AXIS RAPID IP diffractometer
Absorption correction: multi-scan (*ABSCOR*; Higashi, 1995 ▶) *T*_min_ = 0.808, *T*_max_ = 0.887
5866 measured reflections
2062 independent reflections
1275 reflections with *I* > 2σ(*I*)
*R*_int_ = 0.050

#### Refinement

*R*[*F* ^2^ > 2σ(*F* ^2^)] = 0.048
*wR*(*F* ^2^) = 0.135
*S* = 0.99
2062 reflections
138 parameters
H-atom parameters constrained
Δρ_max_ = 0.26 e Å^−3^
Δρ_min_ = −0.30 e Å^−3^

### 

Data collection: *RAPID-AUTO* (Rigaku, 1998 ▶); cell refinement: *RAPID-AUTO*; data reduction: *CrystalStructure* (Rigaku/MSC, 2002 ▶); program(s) used to solve structure: *SHELXTL* (Sheldrick, 2008 ▶); program(s) used to refine structure: *SHELXTL*; molecular graphics: *SHELXTL*; software used to prepare material for publication: *SHELXTL*.

## Supplementary Material

Crystal structure: contains datablocks I, global. DOI: 10.1107/S1600536809031407/xu2586sup1.cif

Structure factors: contains datablocks I. DOI: 10.1107/S1600536809031407/xu2586Isup2.hkl

Additional supplementary materials:  crystallographic information; 3D view; checkCIF report

## Figures and Tables

**Fig. 1.**
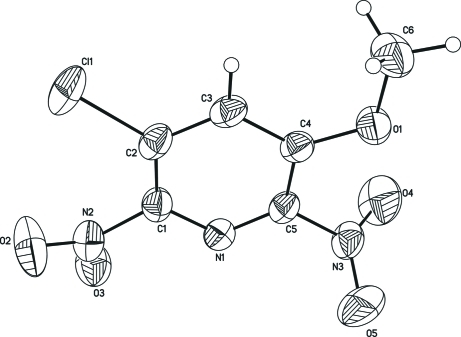
The molecular structure of title compound, with 30% probability displacement ellipsoids (arbitrary spheres for H atoms).

## supplementary crystallographic information

### Comment

Pyridine derivatives are important intermediates used to synthesize pesticide, medicine and play important roles in fine chemical field. 3-Chloro-5-methoxyl- 2,6-dinitro-pyridine was synthesized from 3,5-dichloropyridine N-oxide by substitution and nitration (Bissell *et al.*, 1987), and the process was improved by Chen *et al.* (2008). The crystal structure of the title compound is presented here.

The molecular structure of the title compound is shown in Fig. 1. While the methoxyl group, except H atoms, is co-planar with the pyridine ring, the two nitro groups are twisted with respect to the pyridine ring with dihedral angles of 33.12 (13) and 63.66 (14)°, respectively. Neither hydrogen bonding nor π-π stacking is observed in the crystal structure.

### Experimental

The title compound was prepared according to a literature method (Chen *et al.*, 2008). Crystals suitable for X-ray analysis were obtained by slow evaporation of 1,2-dichloroethane.

### Refinement

H atoms were positioned geometrically and refined using a ride model with C—H = 0.93 Å for aromatic H and 0.96 Å for methyl H atoms, U_iso_(H) = 1.5U_eq_(C) for methyl H atoms and 1.2U_eq_(C) for aromatic H atom.

### Crystal data

**Table:**

C_6_H_4_ClN_3_O_5_	*F*(000) = 472
*M**_r_* = 233.57	*D*_x_ = 1.701 Mg m^−^^3^
Monoclinic, *P*2_1_/*n*	Mo *K*α radiation, λ = 0.71073 Å
Hall symbol: -P 2yn	Cell parameters from 5866 reflections
*a* = 6.6490 (13) Å	θ = 2.5–27.5°
*b* = 10.842 (2) Å	µ = 0.43 mm^−^^1^
*c* = 12.715 (3) Å	*T* = 293 K
β = 95.55 (3)°	Block, colorless
*V* = 912.3 (3) Å^3^	0.50 × 0.40 × 0.28 mm
*Z* = 4	

### Data collection

**Table:**

Rigaku R-AXIS RAPID IP diffractometer	2062 independent reflections
Radiation source: fine-focus sealed tube	1275 reflections with *I* > 2σ(*I*)
graphite	*R*_int_ = 0.050
Detector resolution: 10.00 pixels mm^-1^	θ_max_ = 27.5°, θ_min_ = 2.5°
ω scans	*h* = −8→8
Absorption correction: multi-scan (*ABSCOR*; Higashi, 1995)	*k* = −13→13
*T*_min_ = 0.808, *T*_max_ = 0.887	*l* = −16→16
5866 measured reflections	

### Refinement

**Table:**

Refinement on *F*^2^	Secondary atom site location: difference Fourier map
Least-squares matrix: full	Hydrogen site location: inferred from neighbouring sites
*R*[*F*^2^ > 2σ(*F*^2^)] = 0.048	H-atom parameters constrained
*wR*(*F*^2^) = 0.135	*w* = 1/[σ^2^(*F*_o_^2^) + (0.08*P*)^2^] where *P* = (*F*_o_^2^ + 2*F*_c_^2^)/3
*S* = 0.99	(Δ/σ)_max_ < 0.001
2062 reflections	Δρ_max_ = 0.26 e Å^−^^3^
138 parameters	Δρ_min_ = −0.30 e Å^−^^3^
0 restraints	Extinction correction: *SHELXTL* (Version 4.2; Sheldrick, 2008), Fc^*^=kFc[1+0.001xFc^2^λ^3^/sin(2θ)]^-1/4^
Primary atom site location: structure-invariant direct methods	Extinction coefficient: 0.157 (11)

### Special details

**Table:**

Geometry. All e.s.d.'s (except the e.s.d. in the dihedral angle between two l.s. planes) are estimated using the full covariance matrix. The cell e.s.d.'s are taken into account individually in the estimation of e.s.d.'s in distances, angles and torsion angles; correlations between e.s.d.'s in cell parameters are only used when they are defined by crystal symmetry. An approximate (isotropic) treatment of cell e.s.d.'s is used for estimating e.s.d.'s involving l.s. planes.
Refinement. Refinement of *F*^2^ against ALL reflections. The weighted *R*-factor *wR* and goodness of fit *S* are based on *F*^2^, conventional *R*-factors *R* are based on *F*, with *F* set to zero for negative *F*^2^. The threshold expression of *F*^2^ > σ(*F*^2^) is used only for calculating *R*-factors(gt) *etc*. and is not relevant to the choice of reflections for refinement. *R*-factors based on *F*^2^ are statistically about twice as large as those based on *F*, and *R*- factors based on ALL data will be even larger.

### Fractional atomic coordinates and isotropic or equivalent isotropic displacement parameters (Å^2^)

**Table:**

	*x*	*y*	*z*	*U*_iso_*/*U*_eq_	
Cl1	0.17055 (10)	0.52802 (8)	0.42365 (6)	0.0756 (3)	
O1	0.8010 (3)	0.77739 (16)	0.37270 (12)	0.0536 (5)	
O2	0.2241 (4)	0.3362 (2)	0.27062 (18)	0.0939 (8)	
O3	0.2590 (3)	0.4071 (2)	0.11455 (16)	0.0716 (6)	
O4	0.8115 (4)	0.7839 (2)	0.14363 (19)	0.0949 (8)	
O5	0.9482 (4)	0.6061 (2)	0.1524 (2)	0.1051 (9)	
N1	0.5510 (3)	0.55114 (17)	0.20338 (15)	0.0439 (5)	
N2	0.2845 (3)	0.4126 (2)	0.2107 (2)	0.0590 (6)	
N3	0.8230 (3)	0.6789 (2)	0.17477 (16)	0.0556 (6)	
C1	0.4068 (3)	0.5141 (2)	0.25985 (18)	0.0440 (5)	
C2	0.3749 (3)	0.5646 (2)	0.35677 (18)	0.0453 (6)	
C3	0.5061 (3)	0.6544 (2)	0.39881 (17)	0.0443 (5)	
H3	0.4898	0.6885	0.4645	0.053*	
C4	0.6614 (3)	0.6929 (2)	0.34246 (16)	0.0407 (5)	
C5	0.6705 (3)	0.6373 (2)	0.24397 (16)	0.0412 (5)	
C6	0.7921 (5)	0.8349 (3)	0.47424 (19)	0.0657 (8)	
H6A	0.6641	0.8754	0.4761	0.099*	
H6B	0.8989	0.8944	0.4857	0.099*	
H6C	0.8073	0.7732	0.5286	0.099*	

### Atomic displacement parameters (Å^2^)

**Table:**

	*U*^11^	*U*^22^	*U*^33^	*U*^12^	*U*^13^	*U*^23^
Cl1	0.0570 (4)	0.0974 (6)	0.0777 (5)	−0.0107 (4)	0.0343 (4)	0.0139 (4)
O1	0.0575 (10)	0.0580 (10)	0.0474 (9)	−0.0144 (8)	0.0153 (8)	−0.0112 (8)
O2	0.1109 (18)	0.0831 (15)	0.0882 (15)	−0.0542 (15)	0.0115 (13)	0.0088 (12)
O3	0.0704 (12)	0.0772 (13)	0.0661 (13)	−0.0183 (10)	0.0006 (10)	−0.0065 (10)
O4	0.1047 (18)	0.0908 (18)	0.0946 (16)	−0.0134 (14)	0.0377 (14)	0.0320 (13)
O5	0.0869 (16)	0.1075 (19)	0.133 (2)	0.0047 (15)	0.0717 (15)	−0.0076 (16)
N1	0.0418 (10)	0.0451 (10)	0.0457 (10)	−0.0002 (9)	0.0083 (8)	−0.0001 (8)
N2	0.0494 (12)	0.0609 (14)	0.0666 (15)	−0.0129 (11)	0.0060 (10)	0.0026 (11)
N3	0.0526 (12)	0.0688 (15)	0.0481 (11)	−0.0127 (11)	0.0182 (9)	−0.0061 (11)
C1	0.0400 (11)	0.0430 (12)	0.0492 (12)	−0.0024 (10)	0.0053 (10)	0.0060 (10)
C2	0.0388 (11)	0.0492 (13)	0.0493 (13)	0.0041 (10)	0.0121 (9)	0.0141 (10)
C3	0.0468 (12)	0.0476 (13)	0.0405 (12)	0.0064 (11)	0.0150 (9)	0.0035 (10)
C4	0.0415 (11)	0.0395 (11)	0.0422 (12)	0.0038 (10)	0.0096 (9)	0.0035 (9)
C5	0.0364 (10)	0.0460 (12)	0.0423 (11)	0.0004 (10)	0.0097 (9)	0.0037 (9)
C6	0.0845 (19)	0.0678 (17)	0.0463 (14)	−0.0182 (15)	0.0138 (13)	−0.0125 (12)

### Geometric parameters (Å, °)

**Table:**

Cl1—C2	1.717 (2)	N3—C5	1.476 (3)
O1—C4	1.334 (3)	C1—C2	1.384 (3)
O1—C6	1.440 (3)	C2—C3	1.380 (3)
O2—N2	1.219 (3)	C3—C4	1.378 (3)
O3—N2	1.219 (3)	C3—H3	0.9300
O4—N3	1.206 (3)	C4—C5	1.396 (3)
O5—N3	1.201 (3)	C6—H6A	0.9600
N1—C5	1.300 (3)	C6—H6B	0.9600
N1—C1	1.315 (3)	C6—H6C	0.9600
N2—C1	1.472 (3)		
			
C4—O1—C6	117.90 (18)	C4—C3—H3	120.3
C5—N1—C1	116.89 (19)	C2—C3—H3	120.3
O3—N2—O2	124.9 (2)	O1—C4—C3	126.4 (2)
O3—N2—C1	118.7 (2)	O1—C4—C5	117.78 (18)
O2—N2—C1	116.4 (2)	C3—C4—C5	115.8 (2)
O5—N3—O4	124.5 (2)	N1—C5—C4	126.05 (19)
O5—N3—C5	118.2 (2)	N1—C5—N3	114.31 (18)
O4—N3—C5	117.3 (2)	C4—C5—N3	119.6 (2)
N1—C1—C2	123.3 (2)	O1—C6—H6A	109.5
N1—C1—N2	113.5 (2)	O1—C6—H6B	109.5
C2—C1—N2	123.2 (2)	H6A—C6—H6B	109.5
C3—C2—C1	118.6 (2)	O1—C6—H6C	109.5
C3—C2—Cl1	118.16 (18)	H6A—C6—H6C	109.5
C1—C2—Cl1	123.13 (19)	H6B—C6—H6C	109.5
C4—C3—C2	119.3 (2)		
			
C5—N1—C1—C2	1.9 (3)	C6—O1—C4—C5	−180.0 (2)
C5—N1—C1—N2	−177.3 (2)	C2—C3—C4—O1	−179.3 (2)
O3—N2—C1—N1	−31.5 (3)	C2—C3—C4—C5	1.0 (3)
O2—N2—C1—N1	145.0 (2)	C1—N1—C5—C4	0.9 (3)
O3—N2—C1—C2	149.3 (2)	C1—N1—C5—N3	−177.3 (2)
O2—N2—C1—C2	−34.2 (4)	O1—C4—C5—N1	178.0 (2)
N1—C1—C2—C3	−3.1 (3)	C3—C4—C5—N1	−2.3 (3)
N2—C1—C2—C3	176.0 (2)	O1—C4—C5—N3	−4.0 (3)
N1—C1—C2—Cl1	172.71 (17)	C3—C4—C5—N3	175.7 (2)
N2—C1—C2—Cl1	−8.2 (3)	O5—N3—C5—N1	−64.2 (3)
C1—C2—C3—C4	1.4 (3)	O4—N3—C5—N1	115.0 (3)
Cl1—C2—C3—C4	−174.58 (16)	O5—N3—C5—C4	117.5 (3)
C6—O1—C4—C3	0.3 (3)	O4—N3—C5—C4	−63.3 (3)
